# Lamin B2 binding to minichromosome maintenance complex component 7 promotes non-small cell lung carcinogenesis

**DOI:** 10.18632/oncotarget.20338

**Published:** 2017-08-18

**Authors:** Yinan Ma, Liangru Fei, Meiyu Zhang, Wenzhu Zhang, Xiaofang Liu, Congcong Wang, Yuan Luo, Haiyan Zhang, Yuchen Han

**Affiliations:** ^1^ Departments of Pathology, School of Basic Medical Sciences, China Medical University, Liaoning, China; ^2^ Department of Pathology, The First Affiliated Hospital of China Medical University, Liaoning, China; ^3^ Department of Pathology, Shanghai Chest Hospital, Shanghai, China; ^4^ Department of Pathology, The First People's Hospital of Jining, Shandong, China

**Keywords:** lamin B2, MCM7, DNA replication, NSCLC

## Abstract

We investigated the role of lamin B2 in non-small cell lung cancer (NSCLC). We detected higher lamin B2 expression in 20 NSCLC tumor tissues obtained from The Cancer Genome Atlas than in adjacent normal lung tissues. LMNB2-RNAi knockdown in A549 and H1299 NSCLC cells inhibited colony formation, cell proliferation and G1-S cell cycle progression while increasing apoptosis. LMNB2 overexpression had the opposite effects. Tumor xenograft experiments showed diminished tumor growth with LMNB2 knockdown H1299 cells than with controls. Yeast two-hybrid studies revealed minichromosome maintenance complex component 7 (MCM7) to be a binding partner of lamin B2, which was confirmed by co-immunoprecipitation and co-localization studies. Lamin B2 binding enhanced DNA binding and helicase activities of MCM7. Deletion analysis with MCM7-N, MCM7-M or MCM7-C mutant proteins showed that lamin B2 binds to the C-terminus of MCM7, and competes with the binding of the tumor suppressor retinoblastoma (RB) protein. Immunohistochemical analysis of 150 NSCLC patient samples revealed that both lamin B2 and MCM7 levels positively correlated with histological grade and tumor TNM stage. Moreover, high lamin B2 and MCM7 levels correlated with shorter overall survival of NSCLC patients. In sum, these results show that lamin B2 interaction with MCM7 promotes NSCLC progression.

## INTRODUCTION

Lamins are intermediate filament proteins that form a scaffold known as the nuclear lamina [1]. They are involved in tissue homeostasis and elicit large-scale or fine chromatin conformational changes, accelerate the DNA damage response, and affect transcription factor shuttling [2]. Lamins orchestrate the organization of the genome, which is essential for normal gene transcription and silencing, DNA replication and repair, positioning of nuclear pore complexes, chromatin remodeling, and nuclear envelope breakdown and reassembly during mitosis [3, 4, 5]. DNA replication is stalled in the initiation and elongation phases when the lamin network is disrupted [6, 7]. Lamin mutations are also associated with increased DNA damage [8]. Thus, laminopathies are a heterogenous group of diseases due to lamin mutations and/or defects in their expression or post-translational processing [9, 10].

The mechanical microenvironment plays a crucial role in tumor growth and progression [11, 12]. Since lamins play a central role in the mechanoregulation of gene expression, changes in lamin levels influence cellular response to changes in their mechanical environment [13–16]. There are two types of lamins in mammalian cells, namely, lamin A/C and lamin B, which are encoded by *LMNA*, *LMNB1*, and *LMNB2* genes [17]. Many recent studies have reported link between lamins and cancer. A-type lamins increase the invasiveness of colorectal cancer (CRC) by promoting a more stem cell-like phenotype, thereby decreasing survival times [18]. Low LMNA expression is associated with increased disease recurrence in stage II and III colon cancer patients [19]. Lamin A/C is overexpressed in neuroblastoma, prostate cancer, hepatocellular carcinoma, breast cancer and low grade endometrial cancer [20–25]. Lamin A is aberrantly distributed in the cell line GLC-A1, derived from lung adenocarcinoma [26]. Lamin B1 expression correlates with poor prognosis in hepatocellular carcinoma, prostate cancer and pancrea cancer [27, 28]. However, the role of lamin B2 in cancer is still unclear.

Minichromosome maintenance complex (MCM) components 2–7 are highly conserved from yeast to humans. They are recruited as a hexamer to the chromatin and form the pre-replication complex by binding to the origin recognition complex, Cdt1, and Cdc6 during early G1 phase, thereby initiating DNA replication [29, 30]. To ensure that DNA replication is only initiated once per cell cycle, MCM complexes shut down during S, G2, and early M phase to prevent re-initiation of DNA synthesis [31]. The complex consisting of MCM4, MCM6, and MCM7 has DNA helicase activity [32]. MCM7 is a crucial component of the DNA replication licensing complex in eukaryotes [33] and it might lead to increased or decreased DNA replication licensing activity of the MCM complex, and guide the cells into a higher level of proliferation or cell growth arrest [34]. MCM7 is correlated with tumorigenesis in several human malignancies, including prostate cancer [35], endometrial carcinoma, ovarian cancer, and colorectal adenocarcinoma [36–38]. In this study, we investigated the role of lamin B2 and its association with MCM7 in non-small cell lung cancer (NSCLC).

## RESULTS

### Lamin B2 is overexpressed in NSCLC

**Figure 1:**
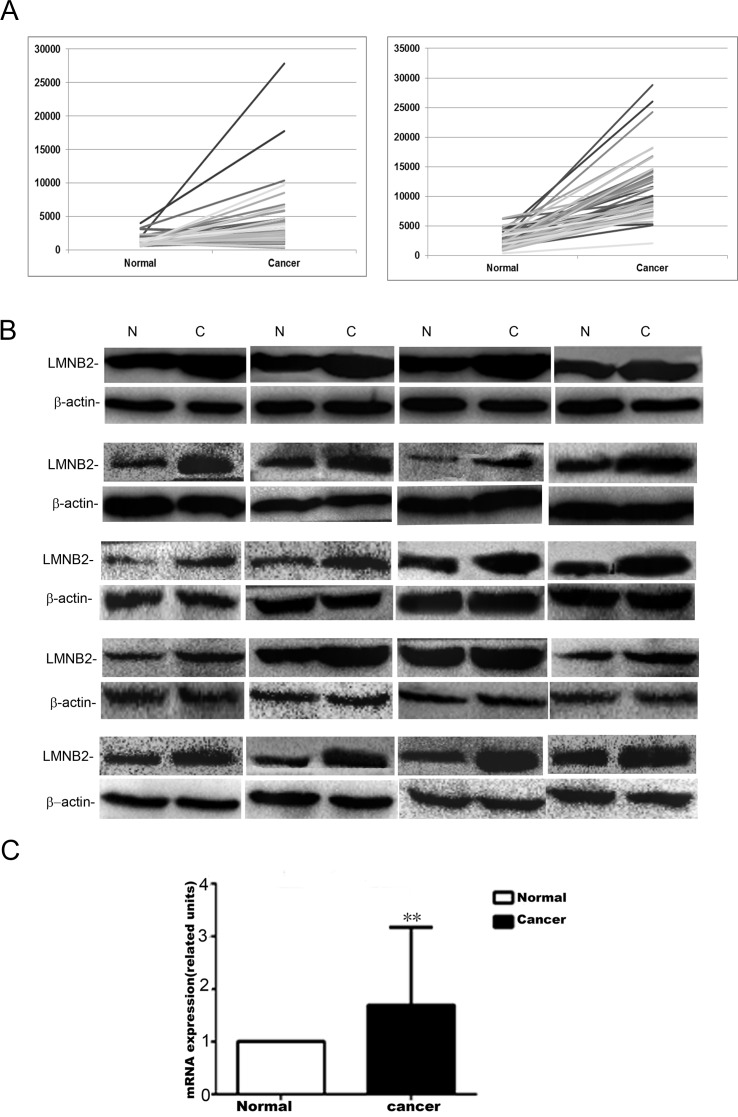
Lamin B2 is overexpressed in NSCLC **(A)** LMNB2 expression in lung cancer samples from the TCGA. LMNB2 is up-regulated in lung adenocarcinoma (left) and in lung squamous carcinoma (right). **(B)** Western blot analysis of lamin B2 protein expression in 20 cases of lung cancer tissues with paired normal lung tissue (p<0.01). β-actin is used as internal control. **(C)** Lamin B2 mRNA expression in 20 cases of lung cancer tissues with paired normal lung tissue (p<0.01).

As shown in Table 1 and Figure 1A, lung cancer samples from the The Cancer Genome Atlas (TCGA) showed high lamin B2 mRNA expression in NSCLC than in normal tissues. Western blot and qPCR analysis also showed high lamin B2 expression in 20 NSCLC cancer tissues than in adjacent normal lung tissues (Figure 1B-1C).

**Table 1:** LMNB2 expression in TCGA lung cancer patient cohorts

Gene	Histology	Sample-up	Sample-down	P -value
LMNB2	LUAD	46	11	1.13E-16
	LUSC	50	0	7.74E-91

1. LUAD: lung adenocarcinoma. LUSC: lung squamous carcinoma. 2. Sample-up: The counts of samples LMNB2 up-regulated in original data (The up-graduated is defined as the cancer sample counts higher than the cancer adjacent sample counts). Sample-down: The counts of samples LMNB2 down-regulated in original data (The down-graduated is defined as the cancer sample counts lower than the cancer adjacent sample counts). 3. P-value<0.05 is significant.

### Lamin B2 promotes lung cancer cell proliferation

**Figure 2:**
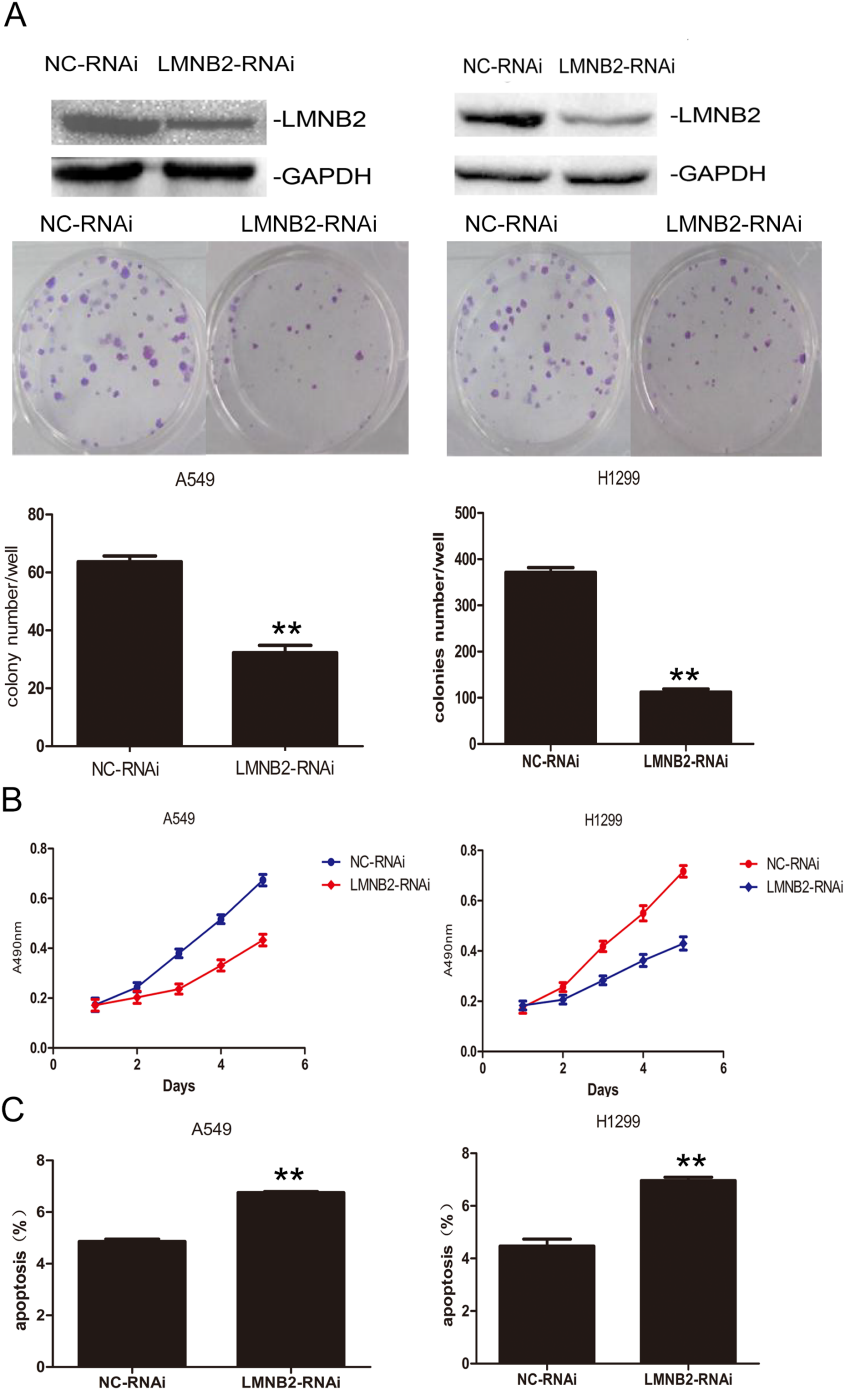
Knockdown Lamin B2 inhibits NSCLC proliferation **(A)** Western blot and colony number analysis of A549 (left) and H1299 (right) NSCLC cells transfected with NC-RNAi and LMNB2-RNAi. **(B)** MTT cell growth analysis of A549 (left) and H1299 (right) NSCLC cells transfected with NC-RNAi and LMNB2-RNAi (P<0.01). **(C)** Apoptotic rates of A549 (left) and H1299 (right) cells transfected with NC-RNAi and LMNB2-RNAi (P<0.01).

**Figure 3:**
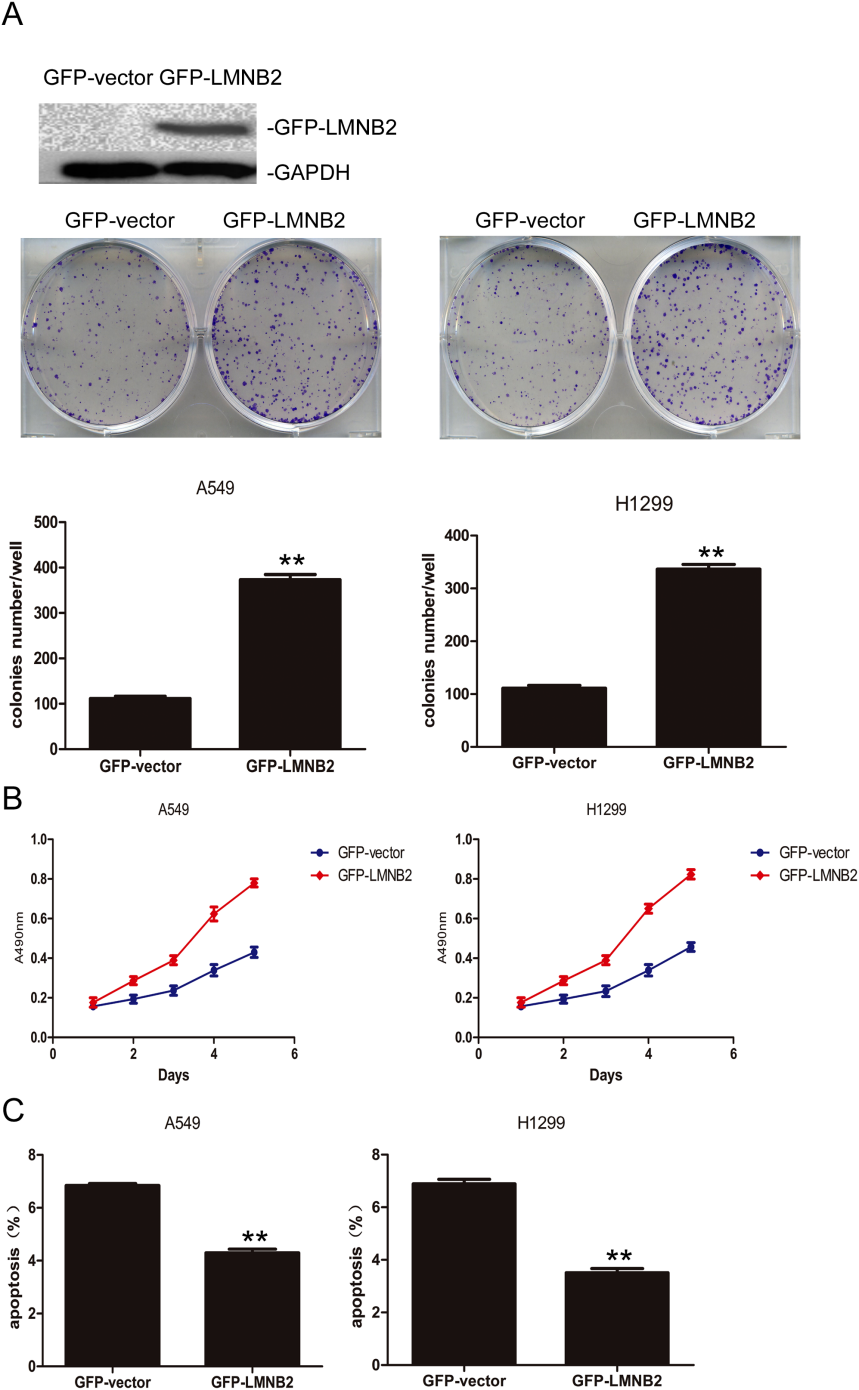
Overexpressed Lamin B2 promotes NSCLC proliferation **(A)** Western blot and colony number analysis of A549 (left) and H1299 (right) NSCLC cells transfected with GFP-vector or GFP-LMNB2 (P<0.01). **(B)** MTT cell growth analysis of A549 (left) and H1299 (right) NSCLC cells transfected with GFP-vector or GFP-LMNB2 (P<0.01). **(C)** Apoptotic rates of A549 (left) and H1299 (right) cells transfected with GFP-vector or GFP-LMNB2 (P<0.01).

**Figure 4:**
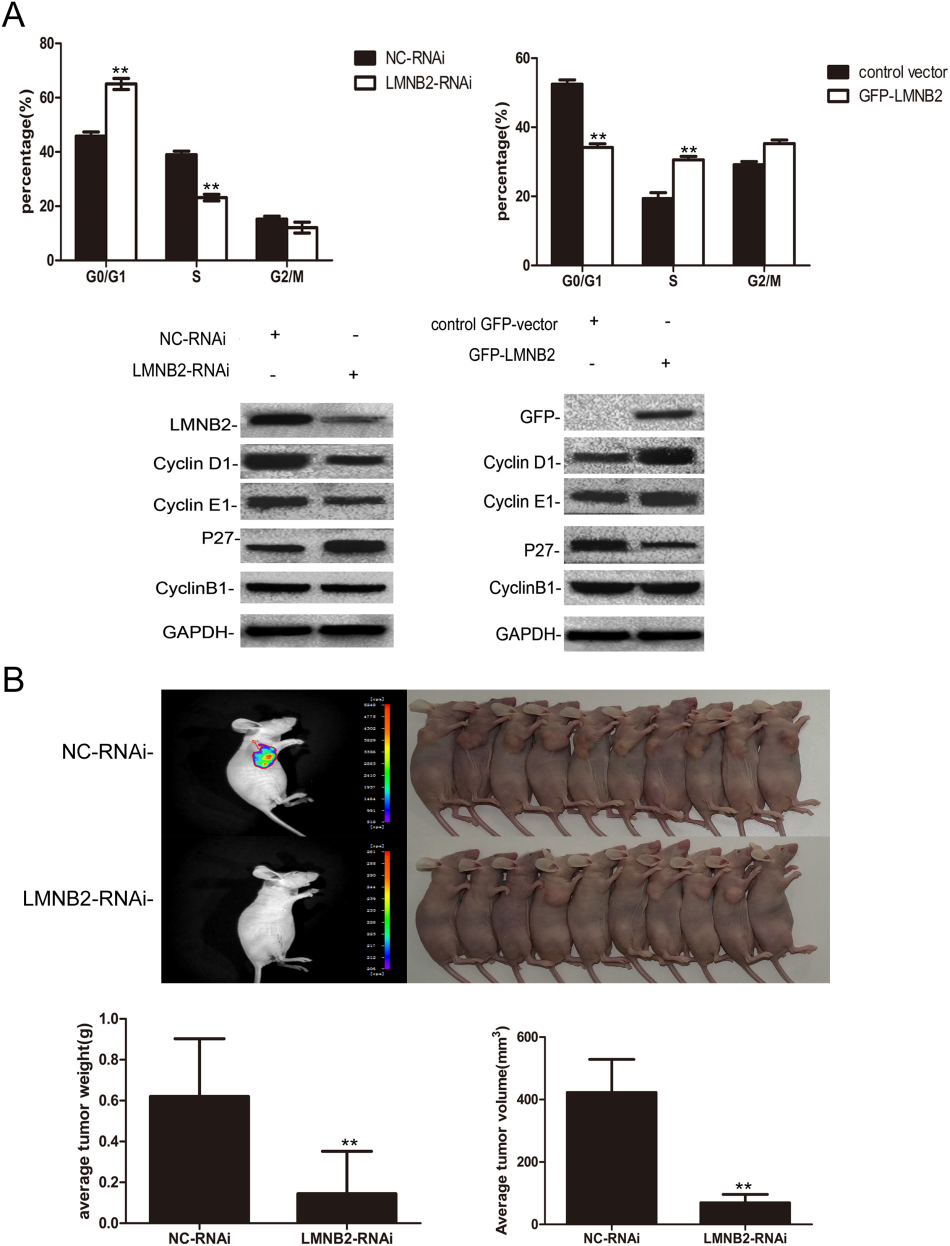
Lamin B2 prolongs S phase and promotes tumorigenesis **(A)** Cell cycle analysis showing percent G0/G1, S and G2/M phase cells in (left) LMB2- or NC-RNAi transfected A549 cells and (right) control GFP vector or GFP-LMNB2 transfected A549 cells. As shown, lamin B2 knockdown increases percent G0/G1 phase cells while decreasing percent S phase cells. On the other hand, overexpression of laminB2 decreases percent G0/G1 phase cells while increasing percent S phase cells. (both P < 0.01). Also, comparative western blot analysis of cell cycle regulatory proteins, cyclin D1, cyclin E1, p27, cyclin B1 in (left) LMB2- or NC-RNAi transfected A549 cells and (right) control GFP vector or GFP-LMNB2 transfected A549 cells is shown. **(B)** BALB/C nude mice (10 mice each, 5 weeks old) were injected subcutaneously with NC- or LMNB2 RNAi transfected H1299 cells in the right flanks. The subcutaneous tumors at 25 days after transplant are shown. Histograms represent average tumor weight (left) and average tumor volume (right) of the mice xenografted with NC- or LMNB2 RNAi transfected H1299 cells (P < 0.01).

To investigate the role of lamin B2 in NSCLC growth, we transiently knocked down LMNB2 with LMNB2-RNAi or overexpressed LMNB2 with GFP-LMNB2 vector in A549 and H1299 lung cancer cell lines with their corresponding controls. LMNB2-RNAi knockdown in NSCLC cell lines inhibited colony formation and cell proliferation, but promoted apoptosis (Figure 2A-2C). Conversely, LMNB2 overexpression increased colony formation and cell proliferation while inhibiting apoptosis (Figure 3A-3C). Moreover, LMNB2-RNAi increased percentage of G0/G1 phase cells (Figure 4A). This suggested that lamin B2 regulated the G1-S checkpoint. To confirm this, we analyzed the effect of lamin B2 knockdown on the regulators of cell cycle progression at the G1/S boundary. LMNB2 knockdown decreased cyclinD1, cyclinE1, and the CDK inhibitor p27 levels whereas cyclinB1, which is involved in G2/M phase were not affected (Figure 4A). Tumor xenograft experiments in nude mice showed that LMNB2 knockdown inhibited tumorigenesis (Figure 4B).

### Lamin B2 binds to MCM7 C- terminus and enhances MCM7 chromatin binding and helicase activities

**Figure 5:**
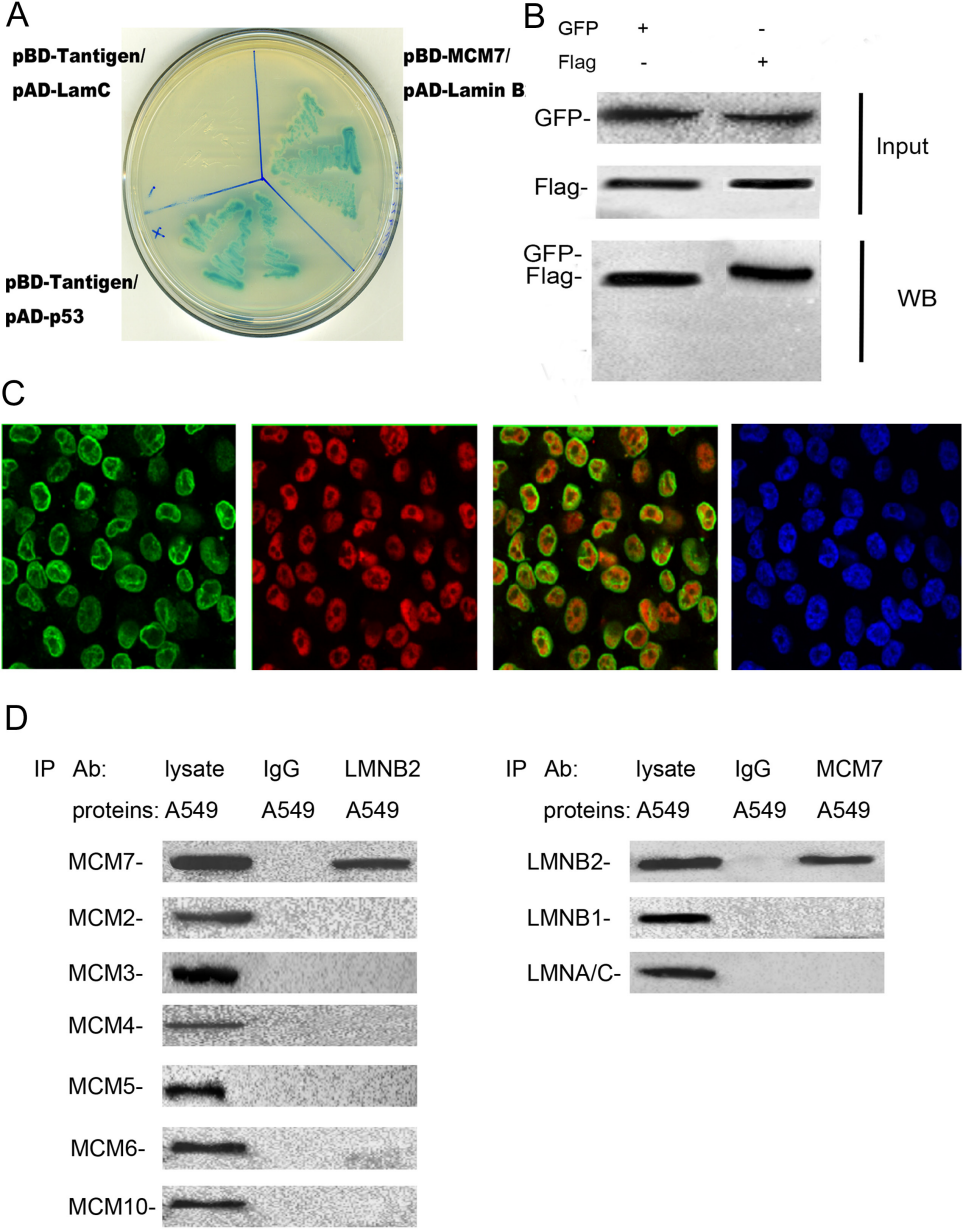
Lamin B2 binds to C-terminus of MCM7 **(A)** The pBD-MCM7 and pAD-Lamin B2 co-transformants were grown on SD agar plates with highly stringent nutrient selection (SD-leu-Trp-His-Ade) confirming the interaction between MCM7 and lamin B2. The pGBKT7-p53 and pGADT7-T-antigen co-transformants were used as positive control and pGBKT7-lam and pGADT7-T-antigen co-transformants were used as negative control. **(B)** Protein lysates of A549 cells that were co-transfected with Flag-MCM7 and GFP-Lamin B2 were immunoprecipitated with anti-GFP (left) or Flag (right) antibodies and immunoblotted with GFP or FLAG antibody. **(C)** Immunofluorescence staining of A549 cells with mouse anti-lamin B2 primary and anti-mouse FITC-conjugated secondary antibodies (green staining) and with rabbit anti-MCM7 primary and anti-rabbit TRITC-conjugated secondary antibodies (red staining). DAPI was used to stain the nuclei (blue staining). **(D)** Co-immunoprecipitation analysis of MCM7 or laminB2 from A549 cell lysates with anti-laminB2 or anti-MCM7 antibodies. IP with anti-IgG antibodies was used as negative control.

Next, we analyzed the interaction between lamin B2 and MCM7. Yeast two-hybrid screening identified lamin B2 as one of proteins that interacted with MCM7. This was confirmed by co-transformation with pBD-MCM7 and pAD-lamin B2 under stringent selection conditions (Figure 5A). Then, we demonstrated lamin B2 interaction with MCM7 *in vivo* by co-transfecting Flag-MCM7 and GFP-Lamin B2 into A549 cells and co-IP experiments (Figure 5B). Moreover, lamin B2 and MCM7 co-localized in the nucleoplasm of A549 cells as visualized by immunofluorescence with anti-lamin B2 and anti-MCM7 antibodies (Figure 5C). Co-immunoprecipitation experiments with anti-lamin B2 or anti-MCM7 antibodies demonstrated direct interaction between lamin B2 and MCM7 (Figure 5D).

**Figure 6:**
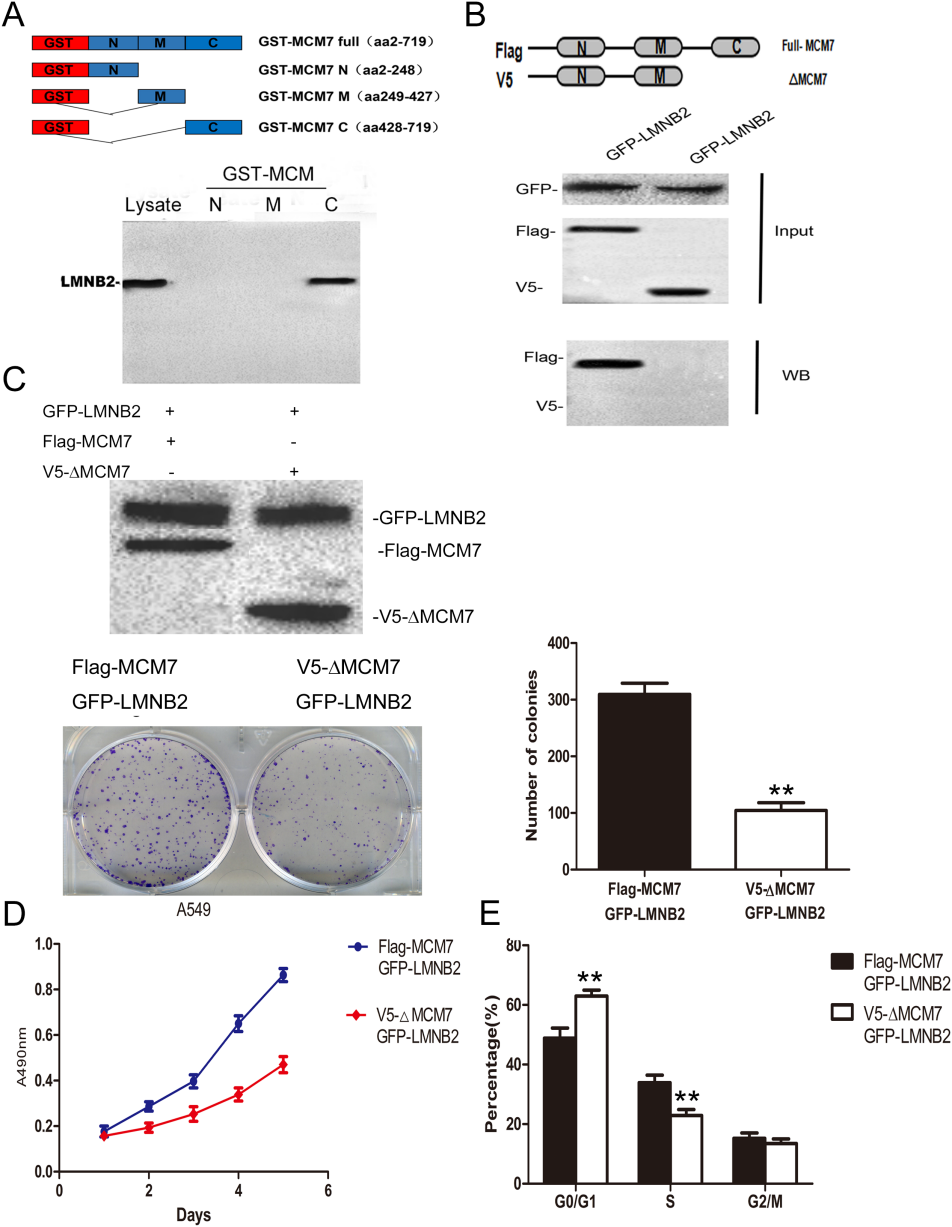
Lamin B2 promotes NSCLC proliferation by interacting with C-terminus of MCM7 **(A)** Analysis of LMNB2 binding domain in MCM7 protein. (Top) Schematic representation of GST tagged full length and different deletion mutants of MCM7, namely MCM7-N, MCM7-M and MCM7-C. (Bottom) LaminB2 binding assays with GST or GST-MCM7 deletion mutants using A549 cell lysates. The bound Lamin B2 was immunoblotted with anti-laminB2 antibodies. **(B)** Confirmation of LMNB2 binding to C-terminus of MCM7. (Top) Schematic representation of FLAG-MCM7 (full length) and V5-ΔMCM7 (C-terminus deletion mutant of MCM7) (Bottom). Co-immunoprecipitation analysis shows that full length FLAG-MCM7 pulls down laminB2 from A549 lysates, V5-ΔMCM7 fails to pull down laminB2, thereby demonstrating that laminB2 binds to C-terminus of MCM7. **(C)** V5-ΔMCM7 transfected A549 cells show diminished colony formation ability than Flag-MCM7 transfected A549 cells (P<0.01) **(D)** MTT cell growth assay shows that V5-ΔMCM7 transfected A549 cells show diminished growth than Flag-MCM7 transfected A549 cells (P<0.01). **(E)** Cell cycle analysis by flow cytometry shows increased percent G0/G1 and decreased percent S phase cells in V5-ΔMCM7 transfected A549 cells than Flag-MCM7 transfected A549 cells (P<0.01).

**Figure 7:**
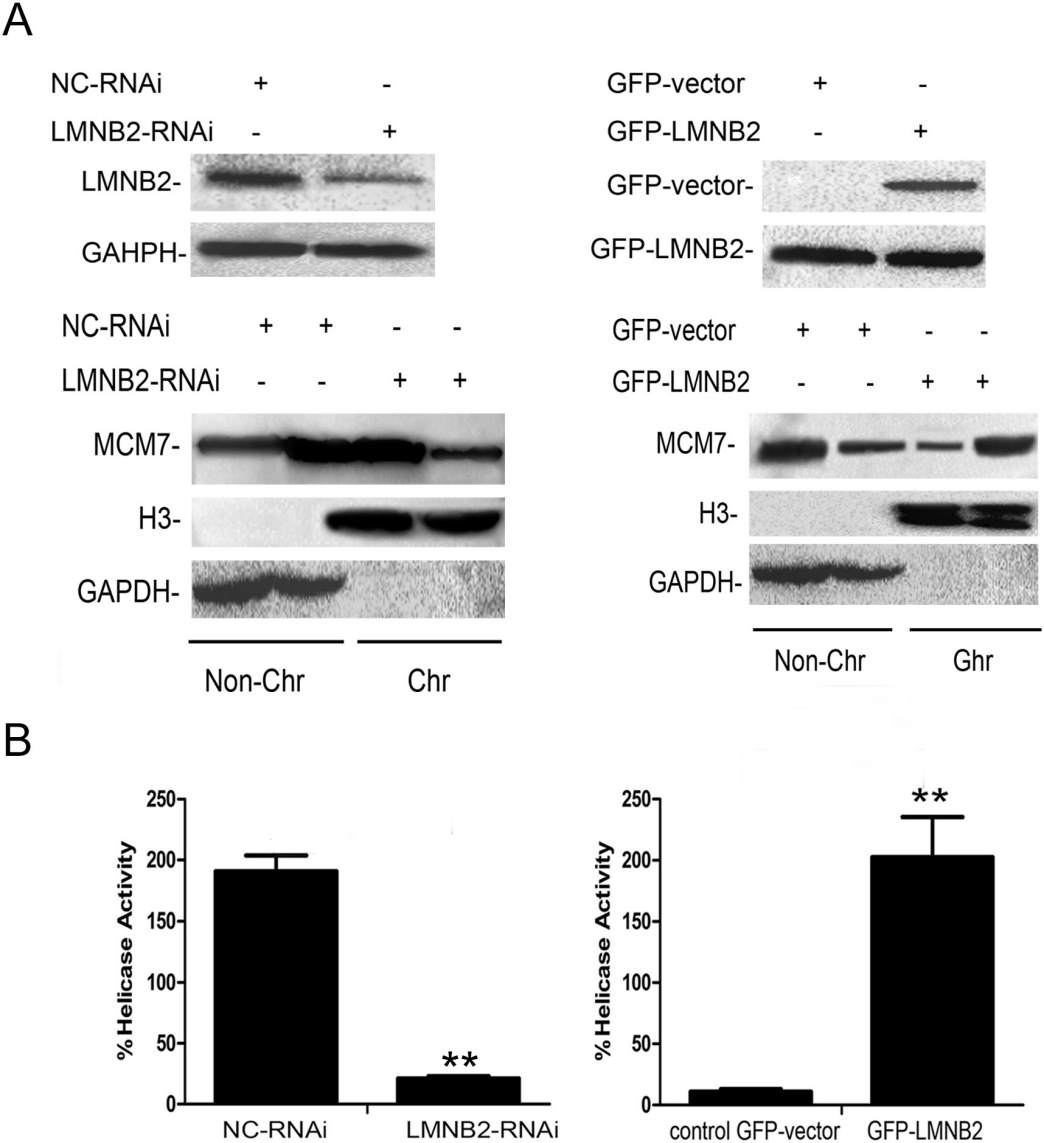
Lamin B2 increases MCM7 activity **(A)** Chromatin association experiments demonstrate that LMNB2 knockdown decreases MCM7 chromatin association, whereas LMNB2 overexpression increases MCM7 chromatin association than in control cells (P<0.01). Note: The chromatin (Chr) and non-chromatin (Non-Chr) fractions were purified and immunoblotted with antibodies specific for MCM7. Antibodies against histone H3 and glyceraldehyde-3-phosphate dehydrogenase were used as chromatin purity controls (P<0.01) **(B)** Helicase activity of MCM7 is decreased in LMNB2-RNAi transfected cells and increased in GFP-LMNB2 transfected cells than in control cells (P<0.01). MCM7 was immunoprecipitated with anti-MCM7 antibodies from A549 cells transfected with shLMNB2 or GFP-LMNB2 vectors and their corresponding controls. Helicase assays were performed with a Cy5-labeled DNA duplex template and biotin-labeled oligocapture probe. The probes were then captured onto streptavidin-agarose beads (Thermo Fisher Scientific, USA) and washed by 1X TBST buffer thrice and analyzed the fluorescence intensity by Thermo Varioskan Flash. (P<0.01).

Next, to identify the binding site for lamin B2 in MCM7, we cloned the N-terminal (247 amino acids), mid-segment (186 amino acids), and C-terminal (219 amino acids) fragments of MCM7 into pGEX-5X to generate GST tagged MCM7-N, MCM7-M, and MCM7-C fusion proteins, respectively (Figure 6A). GST pulldown experiments demonstrated that lamin B2 bound to GST-MCM7 C (Figure 6A). Then, we generated a MCM7 C-terminus deletion mutant, V5-ΔMCM7 and demonstrated by co-immunoprecipitation that lamin B2 binding was abolished by deleting the C-terminal fragment of MCM7 (Figure 6B). Further, transfection of V5-ΔMCM7 into A549 cells decreased colony numbers, cell proliferation and delayed entry into S-phase (Figure 6C-6E). Also, the chromatin binding and helicase activities of V5-ΔMCM7 were inhibited (Figure 7A, 7B). Overall, these data demonstrated that lamin B2 promoted NSCLC proliferation by binding to MCM7 C- terminus and enhancing its activity.

### Lamin B2 competes with RB for binding to the MCM7 C-Terminus

**Figure 8:**
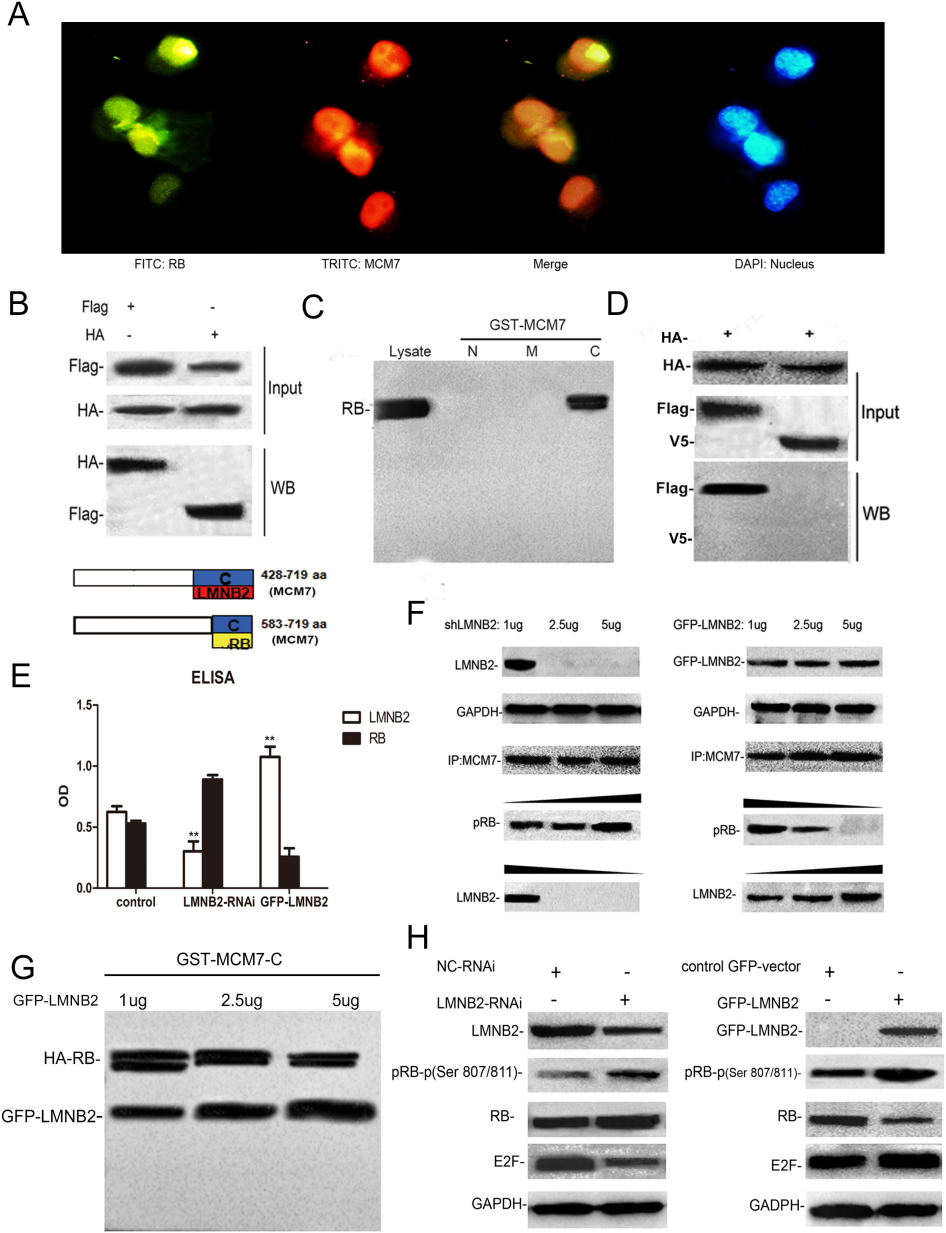
Lamin B2 and RB competitively bind to the MCM7 C-Terminus **(A)** Immunofluorescence staining of A549 cells with mouse anti-RB primary and anti-mouse FITC-conjugated secondary antibodies (green) and rabbit anti-MCM7 primary and anti-rabbit TRITC-conjugated secondary antibodies (red). The nuclei were stained with DAPI. **(B)** MCM7 and RB proteins were immunoprecipitated from lysates of A549 cells transfected with Flag-MCM7 and HA-RB with anti-Flag (left) or HA (right) antibodies. Immunoblotting was performed with anti HA or Flag antibodies, respectively. **(C)** GST pulldown assay with GST-tagged MCM7 deletion constructs namely, MCM7-N, -M and –C shows that RB binds MCM7 C-terminus. **(D)** RB and MCM7 were immunoprecipitated with anti-HA-antibodies from HA-RB/Flag-MCM7 (left) or HA-RB/V5ΔMCM7 (right) transfected A549 cells. The immunoblots were probed with anti-FLAG and anti-V5 antibodies. As shown, RB interacts with the C-terminus of the MCM7. **(E, F)** Co-immunoprecipitation assay shows that increased knockdown (1μg, 2.5μg, 5μg shLMNB2) or overexpression (1μg, 2.5μg, 5μg GFP-LMNB2) of LMNB2 results in increased or decreased binding of RB to MCM7 (P<0.01). **(G)** GST pulldown assay with MCM7-C lysates and different amounts of anti-LMNB2 antibody (1μg, 2.5μg, 5μg) shows decreasing amounts of RB with increasing LMNB2. **(H)** A549 cells transfected with shLMNB2 or GFP-LMNB2 and their corresponding controls shows decreased or increased phospho Ser807/811 RB and E2F, respectively (P<0.01).

The tumor suppressor protein, retinoblastoma protein (RB or pRB) interacts with MCM7 C-terminus region encoding amino acids 583–719 and binds to MCM7 C-terminus 137 amino acids [39, 40]. Immunofluorescence studies demonstrated co-localization of RB and MCM7 in A549 cells (Figure 8A). Also, co-immunoprecipitation showed direct interaction between RB and MCM7 (Figure 8B). GST pull-down assay with MCM7-N, MCM7-M and MCM7-C deletion constructs showed RB binding to the C-terminus of MCM7 (Figure 8C). This was confirmed using the V5-ΔMCM7 transfected A549 cells (Figure 8D). Since both lamin B2 and RB bind to MCM7 C-terminus, we tested if lamin B2 competed with RB for binding to the MCM7 C-terminus. Both co-immunoprecipitation and GST pull down experiments showed competitive binding between RB and lamin B2 for MCM7 (Figure 8E-8G). Besides, we demonstrated increased phospho–Rb and released E2F upon binding MCM7 in A549 cells (Figure 8H).

### High lamin B2 and MCM7 correlate with poor survival of NSCLC patients

**Figure 9:**
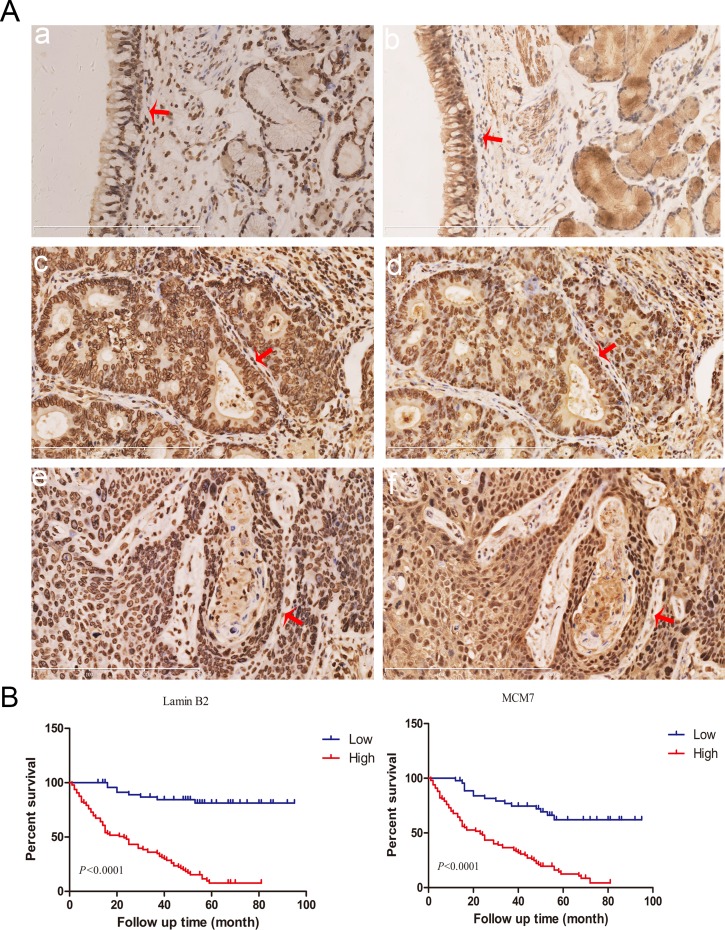
Lamin B2 and MCM7 expression in clinical NSCLC samples **(A)** Immunohistochemical analysis of lamin B2 and MCM7 expression in NSCLC tissue samples. Red arrows point to nuclear laminB2 staining in (a) normal bronchial epithelium, (c) poorly-differentiated lung adenocarcinoma (e) poorly-differentiated squamous cell lung carcinoma and nuclear MCM7 expression in (b) normal bronchial epithelium, (d) poorly-differentiated lung adenocarcinoma, (f) poorly-differentiated squamous cell lung carcinoma. **(B)** Kaplan Meier survival curves show the overall survival rates in 150 NSCLC patients based on low and high laminB2 (left) and MCM7 (right). As shown, high laminB2 and MCM7 expressing patients had poor survival rates than low laminB2 and MCM7 expressing patients.

Next, we performed immunohistochemical analysis of 150 NSCLC patient samples using tissue chips to analyze lamin B2 and MCM7. We observed that lamin B2 levels positively correlated with MCM7 levels (Figure 9A, Table 2). Moreover, both lamin B2 and MCM7 levels correlated with histological grade, and tumor TNM stage (Table 3). A log-rank test showed that high lamin B2 and MCM7 levels correlated with shorter overall survival of NSCLC patients (Figure 9B).

**Table 2:** Correlation between lamin B2 and MCM7 expression in NSCLC patients

lamin B2 Expression	MCM7 Expression					
	Low		High		
	No. patients			R	P
	Low	95	17	0.446	<0.001
	High	15	23		

**Table 3:** Relationship between lamin B2 and MCM7 expression with clinico pathological features in NSCLC patients

			lamin B2			MCM7	
Characteristics	Cases	Low	High	P	Low	High	P
Age							
≤55	27	6(22.2%)	21(77.8%)	0.116	25(29.8%)	59(70.2%)	0.588
>55	123	47(38.2%)	76(61.8%)		17(25.8%)	49(74.2%)	
Gender							
Male	112	38(33.9%)	74(66.1%)	0.537	41(63.1%)	24(36.9%)	0.415
Female	38	15(39.5%)	23(60.5%)		59(69.4%)	46(30.6%)	
Histology							
ADC	75	31(41.3%)	44(58.7%)	0.124	30(31.6%)	65(68.4%)	0.206
SCC	75	22(29.3%)	53(70.7%)		23(41.8%)	32(58.2%)	
Differentiation							
Well	61	40(65.6%)	21(34.4%)	0.002	30(61.9%)	24(38.1%)	0.001
Moderate-Poor	89	35(39.3%)	54(60.7%)		31(35.6%)	56(64.4%)	
TNM stage							
I	71	36(50.7%)	35(49.3%)	0.004	43(68.3%)	20(31.7%)	0.002
I+III+IV	79	22(27.8%)	57(72.2%)		37(42.5%)	50(57.5%)	

ADC: adenocarcinoma; SCC: squamous cell carcinoma.

## DISCUSSION

In this study, we demonstrated that lamin B2 regulates human NSCLC progression by binding to MCM7 C-terminus and enhancing its chromatin binding and helcase activities. Moreover, lamin B2 competes with RB for binding to the MCM7 C-terminus.

Previous studies have demonstrated that lamin A/C is overexpressed in A549 cells and is a potential biomarker for early detection of lung cancer [41]. On the other hand, lamin B1 is a promising biomarker for later stages of lung cancer [42]. However, the relationship between lamin B2 and lung cancer has never been reported. In this study, high lamin B2 expression correlated with differentiation and higher TNM stage of NSCLC. Furthermore, we demonstrated that lamin B2 promotes NSCLC proliferation and tumorigenesis. Lamin B2 interacts with the MCM7 C-terminus, which contains the helicase catalytic domain, whereas the N-terminal region participates in DNA binding [43–45]. Lamin B2 binding to MCM7 C-terminus enhances the catalytic domain and promotes the DNA association and helicase activity. MCM7 C-terminus contains two domains, carboxy-terminal domain (CTD) and subunit-specific sequence extensions at their C termini (CTE) [46]. Moreover, lamin B2 competes with RB for binding the MCM7 C-terminus. When RB binds MCM7, it suppresses the helicase activity of MCM7 [40]. Therefore, we postulate that lamin B2 binding to MCM7 competes out RB and promotes MCM7 helicase activity and subsequent cell proliferation.

RB is a chromatin-associated protein that limits the transcription of cell cycle genes primarily by regulating the E2F transcription factor. RB recruits and stabilizes complexes that repress transcription of E2F targets, thereby restricting the expression of genes required for cell proliferation [47, 48]. RB is broadly expressed and its activity is controlled by cyclin-dependent kinases (CDKs) [49]. MCM8 bound cyclin D1 and activated Rb protein phosphorylation by cyclin-dependent kinase 4 (CDK4) *in vitro* and *in vivo*. The cyclin D1/MCM8 interaction is required for Rb phosphorylation and S-phase entry in prostate cancer cells [50]. RB undergoes selective phosphorylation by p38 in its N-terminus and becomes insensitive to the inactivation by CDKs [51]. Above all, the N-terminus of RB is critical for CDK phosphorylation. We showed that RB binds to MCM7 near the phosphorylation target sites of CDK and thereby decreases RB phosphorylation. Also, we demonstrated that when lamin B2 competes with RB for binding MCM7, RB is hyper-phosphorylated resulting in release of E2Fs. These results confirm the competitive binding of lamin B2 and RB to MCM7. On the contrary, it is possible that since lamin B2 promotes NSCLC cell proliferation and DNA replication, it may consequentially lead to inactive RB and release E2Fs.

Lamin B2 is a biomarker of novel proteins involved in chromosomal instability [52]. The loss of lamin B2 relieves the spatial positional constraints required to maintain the conserved localization of aneuploid chromosome territories in the interphase nucleus [53]. In ovarian cancer, lamin B2 is overexpressed [54], whereas its low expression in prostate cancer correlates with lymph node metastasis [55]. In our study, high lamin B2 increases NSCLC cell proliferation.

Recently, there were some studies about the significant role of protein-protein interactions in biological systems relevant to various human diseases, such as: a rhodium(III) complex emerged as a potent inhibitor of STAT3 that targeted the SH2 domain and inhibited STAT3 phosphorylation and dimerization. Then, the complex exhibited potent anti-tumor activities in an *in vivo* mouse xenograft model of melanoma [56]. Metal-based complex 1 as a direct inhibitor of TLR1/2 heterodimerization [57]. In our study, we found that lamin B2 interacted with MCM7 then promoted NSCLC proliferation.

In summary, we demonstrate that lamin B2 promotes NSCLC growth and progression by competitively binding to MCM7 and dislodging RB, thereby enhancing chromatin binding and helicase activities of MCM7.

## MATERIALS AND METHODS

### NSCLC cell culture and transfections

Human lung cancer cell lines A549 and H1299 were purchased from the Type Culture Collection of the Chinese Academy of Sciences (Shanghai, China). They were grown at 37°C in 5% CO_2_ in RPMI-1640 medium supplemented with 10% fetal bovine serum (Gibco, Life Technologies) and 1% penicillin-streptomycin (JRH Biosciences, St. Louis, MD, USA).

The pCMV6-LMNB2-GFP and control pCMV6-GFP vectors were purchased from OriGene Technologies (Beijing, China). The pENTER-MCM7-Flag and pENTER-ΔMCM7-V5 were purchased from Taihegene Technologies (Beijing, China). The shRNA targeting the *LMNB2* (sense, 5′-TGGAGATCAACGCCTACCG-3′; antisense, 5′-AGCCGCTTCCGCTTACTG-3′) were designed by the Shanghai GeneChem, Co. Ltd, China. The lentiviral vector containing LMNB2 shRNA was named LMNB2-RNAi, whereas the vector containing scrambled control shRNA was named NC-RNAi. Transfection with plasmids, shRNA and GFP-vector into A549 and H1299 cell lines were performed using Lipofectamine 3000 (Invitrogen, USA).

### Patients and specimens

This study was approved by the Human Research Ethics Committee of China Medical University. All patients gave informed consent and were subject to close follow-up observations. None of these patients received chemotherapy or radiotherapy before the operation. Histological grade, histological type and lymphatic metastasis were determined by the pathologists. Tumor stages were classified according to the tumor node metastasis (TNM) classification of the American Joint Committee on Cancer and the International Union. The primary surgical resection tumor specimens were from the collection of the First Affiliated Hospital of China Medical University, Department of Pathology, March 2011 - December 2012, including 4 primary tumor and matched normal lung tissue samples.

### Western blotting

The cell lysates were suspended in RIPA buffer (Millipore, Billerica, MA, USA) and equal amounts (50 μg) of total protein samples were loaded onto an 8% SDS-PAGE gel and electrophoresed at 100V for 1.5h. The separated proteins were transferred onto PVDF membrane (Millipore, Billerica, MA, USA) at 100V for 1 h. The membranes were then blocked with 5 % BSA in 1X TBST for 2 h at room temperature followed by incubation with the primary antibodies overnight: Lamin B2 (Abcam, ab8983), Lamin A/C (Abcam, ab169532) and Lamin B1 (Abcam, ab16048). MCM2 (Proteintech, 10513-1-AP), MCM3 (Proteintech, 15597-1-AP), MCM4 (Proteintech, 13043-1-AP), MCM5 (Proteintech, 11703-1-AP), MCM6 (Proteintech, 13347-1-AP), MCM7 (Proteintech, 11225-1-AP), MCM10 (Abcam, ab3733). Phospho-Rb (Ser807/811) (Cell Signaling Technology, #8516), RB (Wanlei Bio, WL01884), E2F (Wanlei Bio, WL02394). After washing thrice with 1X TBST, the membranes were incubated with the secondary antibodies for 1 h at room temperature. Secondary antibodies: Goat Anti-Mouse IgG H&L (HRP) (Abcam, ab6789) and Goat Anti-Rabbit IgG H&L (HRP) (Abcam, ab6721). All the primary and secondary antibodies were diluted 1:1000. Then, the blots were developed and visualized using the ECL chemiluminescence western blot kit (Thermo Fisher Scientific, USA).

### Colony formation assay

The A549 and H1299 cells (1000 cells/dish) were grown in 40 mm dishes for 24 h after transfection, and incubated for 12 days to develop single cell derived colonies. RPMI medium with 10% FBS was changed every 4 days. On day 12, the plates were washed with PBS and stained with hematoxylin and the number of colonies with more than 50 cells was counted.

### MTT cell growth assay

3-5 × 10^3^ cells were plated in 96-well plates, 24 h after transfection. Then, 20 μl of 5 mg/ml 3-(4,5-Dimethylthiazol-2-yl)-2,5-diphenyltetrazolium bromide (MTT) was added to each well and the plates were further incubated for 4 h at 37°C. The media was removed followed by addition of 200 μl DMSO to dissolve the deposits. The samples were analyzed in an automatic microplate reader at 490 nm. The measurements were performed every 24 h for 5 days and a cell growth curve was generated from the data.

### Annexin V and propidium iodide (PI) staining for apoptosis assay

Apoptosis was assessed via flow cytometric analysis of NC-RNAi/LMNB2-RNAi or GFP-vector/GEP-LMNB2 treated A549 or H1299 cells that were stained with FITC-Annexin V and PI using the Annexin V-FITC apoptosis detection kit according to the manufacturer's protocol (BD Bioscience). Cells were seeded onto 6 well plates and allowed to adhere. After cells become 70% of confluent, cells were treated with NC-RNAi/LMNB2-RNAi or GFP-vector/GEP-LMNB2 for 48 hours at 37°C and 5% CO2. Subsequently, the cells were collected, washed in PBS and resuspended in 500 μl of 1X Annexin-binding buffer. Cells were then incubated at room temperature with Annexin V-FITC and PI stain in the absence of light. Following the 10-minute incubation, samples were immediately analyzed via flow cytometry. Annexin V staining was detected as green fluorescence and PI as red fluorescence.

### Cell cycle analysis by flow cytometry

A549 cells were synchronized at G0/G1 phase by serum starvation. These cells were transfected with control shRNA or shLMNB2 or control GFP-vector or GFP-LMNB2. After 48 h, the cells were fixed with 70% ethanol overnight at 4°C and then washed with PBS containing 1% BSA. The cells were then incubated with 50 μg/ml propidium iodide and 100 μg/ml RNase A in PBS for 30 min followed by flow cytometry analysis in a FACS Calibur (BD Biosciences). 10000 cells were analyzed per sample.

### Co-immunoprecipitation

Cells were washed with chilled PBS and solubilized in lysis buffer containing NP-40 and 1% PMSF. Cell lysates were clarified by centrifugation at 15,000 x*g* for 25 mins at 4°C. Then, 1 mg total protein lysates were incubated with 5 μg Lamin B2 (Abcam, ab8983) or MCM7 (Proteintech, 11225-1-AP) at 4°C overnight with constant rotation. Then, 50 μl of 50% protein G sepharose slurry was added to the mixture and further incubated for 4h with constant rotation. The beads were then collected and washed with PBS and then subjected to western blotting as described above.

### Yeast two hybrid

The fusion protein pBD-MCM7 contained 719 amino acids from MCM7 and 219 amino acids from bait domain [58]. The construct was transformed into One ShotTM competent cells (Invitrogen, Carlsbad, CA). The pAD-LMNB2 vector was generated from pACT2 in 0.5 ml of polyethylene glycol/LiAc at 30°C for 30minutes. After this initial incubation with plasmid DNA, the cell solution was combined with 20 mL of DMSO and incubated for 15 minutes at 42°C. The cells were pelleted, resuspended in 1 mL YPD medium, and shaken at 30°C for 40 minutes. The transformed cells were then pelleted, resuspended in 0.5 mL 0.9% NaCl, and plated onto the appropriate SD agar plate. The transformants were first plated on low stringency SD-Leu/−Trp and medium stringency SD-Leu/−Trp/− His plates. The colonies that grew on those plates were then subjected to the β-galactosidase assay as previously described for 24 and then allowed to grow further on the high stringency SD-Ade/−His/−Leu/−Trp plate.

### GST fusion proteins and GST pull down

The Escherichia coli cells harboring pGST-MCM7 mutants or pGST were grown in 100 ml Luria-Bertani medium supplemented with 100μg/ml ampicillin overnight. They were then induced by 1mM IPTG (Thermo Fisher Scientific, USA) for 3 h. The cells were pelleted, resuspended in 1×PBS, and sonicated for 2 min. The proteins were solubilized in 1% Triton X-100. The supernatant was collected after centrifugation at 15,000g for 5 min. The GST, GST-MCM7N, GST-MCM7M, GST-MCM7C mutant fusion proteins were purified through a glutathione-Sepharose 4B column (Amersham Bioscience). The pull-down assays were analyzed by western blot as previously described.

### Chromatin association assay

Cell lysates were resuspended in 1 ml Buffer A (110 mM KC_2_H_3_O_2_, 15mM NaC_2_H_3_O_2_, 2 mM MgC_2_H_3_O_2_, 0.5 mM EGTA, 20 mM HEPES pH 7.3). The cell suspension was incubated at 4°C for 10 min in a rotator with 2 mM DTT and 50 μg/ml digitonin. Nuclei were pelleted by centrifugation at 1500 x*g* for 10 min and resuspended in hypotonic buffer B (1mM HEPES pH 7.5, 0.5 mM EDTA and 0.5% NP-40). The nuclear suspensions were then incubated at 4°C for 15 min in a rotator and laid on top of a 10 ml sucrose cushion (100 mM sucrose in 0.5 mM Tris-HCl, pH 8.5) and centrifuged at 3500 x*g* for 15 min at 4°C. The chromatin pellets were resuspended in 0.25 mM EDTA pH 8.0, and sonicated thrice for 10 s. The chromatin suspensions were centrifuged twice at high speed for 5 min at 4°C and the supernatants were retained for western blot analysis.

### Helicase activity assay

The immunoprecipitated MCM7 was incubated with a PCR product of a pUC19 template generated with 5′-CAAGTTGGGAAGACAACCTG-3′ and 5′-Cy5-CCAATATGGTGAAACCCCGT-3′ primers, 20 mM Tris-HCl, pH 7.4, 50 mM NaCl, 3 mM MgCl_2_, 2 mM ATP, 20% glycerol, 0.1% bovine serum albumin, and capture probe, 5′-CAAGTTGGGAAGACAACCTGTAGGGCCTGCGGGGT-3′ for 30 minutes at room temperature. The reaction was stopped by adding 170 mM EDTA, pH 8.0. The probes were then captured onto streptavidin-agarose beads (Thermo Fisher Scientific, USA) and washed by 1X TBST buffer thrice and analyzed the fluorescence intensity by Thermo Varioskan Flash.

### Nude mice NSCLC xenograft protocol

H1299 cells were stably transfected with either NC-RNAi or LMNB2-RNAi. Twenty 5-week old BALB/C nude mice (SLAC Laboratory, Shanghai, China) were subcutaneously injected with 100 μl control or LMNB2 knockdown H1299 cells (3×10^6^ cells mixed with matrigel in a 1: 1 ratio) into right flanks. After 25 days, the tumor volume and weight were recorded every two days until the 15th mice were sacrificed. The mice experiments were performed according to the protocols approved by the Institutional Animal Care and Use Committee.

### Statistical analysis

Immunohistochemistry results were analyzed using the chi-square test and Spearman rank correlation. Kaplan- Meier survival analyses were carried out and compared using the log-rank test. Differences between groups were compared using two-tailed Student's t-test; p values < 0.05 (^*^) or < 0.01 (^**^) were considered statistically significant.

## Abbreviations

NSCLC: non-small cell lung cancer
MCM7: Minichromosome maintenance complex component 7
